# Newly Developed TV-Based Cognitive Training Games Improve Car Driving Skills, Cognitive Functions, and Mood in Healthy Older Adults: Evidence From a Randomized Controlled Trial

**DOI:** 10.3389/fnagi.2019.00099

**Published:** 2019-05-07

**Authors:** Rui Nouchi, Akiko Kobayashi, Haruka Nouchi, Ryuta Kawashima

**Affiliations:** ^1^Department of Cognitive Health Science, Institute of Development, Aging and Cancer (IDAC), Tohoku University, Sendai, Japan; ^2^Smart Aging Research Center (S.A.R.C.), Tohoku University, Sendai, Japan; ^3^Department of Functional Brain Imaging, Institute of Development, Aging and Cancer (IDAC), Tohoku University, Sendai, Japan

**Keywords:** car driving skill, cognitive training, processing speed, inhibition, positive mood

## Abstract

**Background**: Cognitive training in a laboratory improves car driving skills of older car drivers. However, it remains unclear whether other types of cognitive training at home have beneficial effects on driving skills. Using our developed cognitive training games that can be played on a television with a set-top box in a person’s home, we investigated the effects of a 6-week cognitive training program on driving skills, which included on-road evaluation (primary outcome), and cognitive functions and emotional states (secondary outcome) in older people.

**Methods**: In this double-blinded randomized control trial (RCT), 60 older licensed drivers were randomly assigned into one of the two groups: a cognitive training game for car driving (CTCD) group and an active control cognitive training game (ACT) group. Participants in the CTCD group played the CTCD (processing speed, dual attention, and speed prediction) for 20 min in five sessions per week for 6 weeks. Participants in the ACT group played the ACT (selecting the larger number; selecting a number from largest to smallest; play a game of rock, article, scissors) for 20 min in five sessions per week for 6 weeks. We measured driving skills, various cognitive functions, and emotional states before and after the 6-week intervention period.

**Results**: Our main results showed that compared to the ACT group, the CTCD group demonstrated improved driving skills (adjusted *p* = 0.034). Moreover, the CTCD group demonstrated improved inhibition (stroop, adjusted *p* = 0.042: reverse Stroop, adjusted *p* = 0.043) and processing speed performance symbol search (SS), adjusted *p* = 0.049; digit symbol coding (adjusted *p* = 0.047), compared to the ACT group. The CTCD group scored higher on vigor–activity mood (adjusted *p* = 0.041) as measured using the Profile of Mood State.

**Discussion**: This randomized controlled trial provides scientific evidence for the benefits of the 6-week CTCD program on driving skills and cognitive functions, such as processing speed, inhibition, and vigor–activity mood, in healthy older people. Our results suggest that cognitive training is useful to improve the driving skills of older adults.

**Trial registration**: This trial was registered at The University Hospital Medical Information Network Clinical Trials Registry (UMIN 000029769). Registered 31 October 2017, https://upload.umin.ac.jp/cgi-open-bin/ctr_e/ctr_view.cgi?recptno=R000034010

## Background

An increase in the aging population has led to an increase in the number of older car drivers worldwide. There is also an increasing risk of car accidents among older adults. Recent road safety studies have typically reported that younger and older car drivers have higher fatal and non-fatal crash risks than the middle-aged car drivers (McAndrews et al., 2013; Ma and Yan, 2014). In addition, previous studies have indicated that older car drivers experience a higher number of car accidents per unit distance traveled than other age groups (Li et al., 2003; Lombardi et al., 2017). Age-related cognitive and physical functional changes are a main factor of car accidents in the older adults (Anstey et al., 2012). Normal aging shows declines in several cognitive abilities needed for driving (Boot et al., 2014). Earlier reports have described that declines of processing speed, attention, and executive functions affect safe driving in older adults (Anstey et al., 2005; Horikawa et al., 2009; Adrian et al., 2011). In fact, car driving skills of older adults were lower than that of younger adults (Doroudgar et al., 2017). Nevertheless, driving is an important activity for older adults to maintain their mental health (Edwards et al., 2009a) and social relationships in a community (Dickerson et al., 2007). Older car drivers want to drive and possess a driver’s license (Yassuda et al., 1997). Thus, researchers have shown great interest in developing ways to improve driving skills and reduce crash risks associated with older drivers.

There are several approaches, such as in-class and on-road education program, physical training, simulator-based training, and cognitive training, to improve the driving skills of older people (Karthaus et al., 2016). The first approach is a post-license educational program, by which older drivers receive in-call or on-road education (Stalvey and Owsley, 2003; Owsley et al., 2004). Such post-license educational programs can increase awareness and reduce exposure to risky driving situations (Boot et al., 2014). The second approach is a physical training program to improve car driving skills. A previous meta-analysis reported that physical functions are associated with driving skills in older adults (Mielenz et al., 2017). Therefore, it is expected that physical training would improve the car driving skills. For example, the 8-week joint-range-of-motion physical fitness training program was shown to improve car driving skills (Ostrow et al., 1992). Another study reported that a 12-week strength exercise training program improved car driving skills, measured by on-road evaluation (Marottoli et al., 2007). However, such education and physical training programs are not easy to conduct, because these training programs need specific trainers (e.g., car driving instructors and psychical therapist). The third approach is a simulator-based training. Several studies demonstrated that car driving simulator intervention improved car driving skills and cognitive functions in the older adults (Casutt et al., 2014b). The advantage of a simulator-based training is that participants can receive highly controlled, repeated training with immediate feedback. However, for older adults, a potential disadvantage of driving simulators is simulator sickness. A recent study revealed that older adults are at a high risk for simulator sickness (Matas et al., 2015). Simulator sickness is a risk factor for the dropout in studies. The fourth approach—cognitive training—involves cognitive training programs designed to enhance cognitive functions and daily behaviors through training tasks and games related to cognitive functions. Earlier studies have reported that a decline in cognitive abilities is associated with a decline in driving ability (Boot et al., 2014). For example, processing speed and executive function are important for the driving performance of older adults (Anstey et al., 2005; Horikawa et al., 2009; Adrian et al., 2011). Therefore, cognitive training is expected to enhance driving skills by improving cognitive abilities such as processing speed and executive functions. Earlier studies have demonstrated improved driving skills and cognitive functions in healthy older adults after cognitive training (Nozawa et al., 2015).

In this study, we focused on cognitive training because it is easy and safe to administer compared to the other approaches. Some previous studies have reported positive effects of cognitive training on driving skills (Roenker et al., 2003; Edwards et al., 2009a,b; Boot et al., 2014; Ross et al., 2018). Nevertheless, some unclear issues remain. First, cognitive training for driving skills has been conducted mainly with trainers (or experimenters) or specific devices or using simulators at laboratories (Casutt et al., 2014a, 2016; Nozawa et al., 2015; Hay et al., 2016; Haeger et al., 2018). To reduce costs related to cognitive training and to extend the outcomes of the cognitive training to the society at large, it is necessary to develop new training methods that can be easily carried out at home. Second, cognitive training programs in driving studies have traditionally used a single-domain cognitive training such as visual processing speed training, like Useful Field of View (UFOV) training (Ball et al., 1988), and attention training (Casutt et al., 2016). Generalization of the effects of cognitive training on driving skill warrants an investigation of whether other types of cognitive training can improve driving skills. Third, previous cognitive training studies have usually used a no-intervention/no-training group for comparison (Ball et al., 2002; Haeger et al., 2018); alternatively, they have used other training programs with different devices (Nozawa et al., 2015; Casutt et al., 2016). Such a study design cannot exclude placebo effects or other potential factors such as learning new devices and meeting new people. Moreover, previous studies have not conducted an randomized control trial (RCT) based on the CONSORT guidelines (Schulz et al., 2010). An RCT with an active control group should be used for comparison in order to obtain evidence for the effects of cognitive training on driving skills.

In this study, to enhance driving skills, we developed cognitive training games that can be used with a television (TV)—the most popular home device. Older adults watch more TV programs than young adults (Mundorf and Brownell, 1990). In fact, watching TV is the most common leisure activity among older adults (Horgas et al., 1998; Strain et al., 2002). Previous cognitive training studies using a TV reported improved cognitive functions in older adults (Shatil et al., 2014). Participants can play all cognitive training games on their own TV at home. There were three cognitive training games (processing speed, dual attention, and speed prediction), because the results of the previous studies indicated that training for multiple domains or tasks had better benefits than did those that included a single domain or task training (Cheng et al., 2012; Nouchi et al., 2012a, 2013). In this study, we selected processing speed, dual attention, and speed prediction tasks as the cognitive training games for the following reasons. Driving is a complex and dynamic activity that involves various cognitive functions. It is difficult to conclude the core cognitive function involved in driving. However, existing evidence shows that processing speed, dual attention, and speed prediction are important for car driving performance. First, previous studies suggest that processing speed, divided attention, and speed prediction scores are correlated with driving skills (Maruyama and Kitamura, 1961; Anstey et al., 2005; Clay et al., 2005; Horikawa et al., 2009; Adrian et al., 2011; Cuenen et al., 2015). Second, cognitive training studies using processing speed, divided attention, and executive functions demonstrated improvements in driving skills and cognitive functions (Roenker et al., 2003; Nozawa et al., 2015; Ross et al., 2018). Based on these findings, we developed a cognitive training program, which included processing speed, dual attention, and speed prediction.

Additionally, we measured the emotional state of the participants before and after the intervention period. It has been reported that subjective emotional states are correlated with driving skills (Matthews et al., 1996; Garrity and Demick, 2001; Chliaoutakis et al., 2002). Furthermore, cognitive training can alter emotional states (Takeuchi et al., 2014; Nouchi et al., 2016a). Therefore, it is important to investigate whether cognitive training for driving skills can engender change in the emotional states and examine the relationship between change in emotional states and improvement in driving skills.

The main purpose of this trial was to investigate the benefits of TV-based cognitive training on driving skills in a healthy aging population. Therefore, we conducted a 6-week double-blinded RCT with two parallel groups: a cognitive training game for car driving skill (CTCD) group and an active control cognitive training game (ACT) group. To evaluate the effects of the developed cognitive training game, we assessed driving skills using an on-road evaluation test, cognitive functions, and emotional states. The primary outcome was driving skill performance. Based on previous studies (Wolinsky et al., 2013; Nozawa et al., 2015; Ball et al., 2002), we expected that the developed cognitive training games would engender improvements in driving skills, cognitive function, and emotional states of the CTCD group compared to the ACT group.

## Method

### Randomized Controlled Trial Design and Setting of This Trial

This RCT was conducted from November 2017 to January 2018 in Sendai, Shiogama, and Kurihama cities, Japan. The population of each city in 2018 was 1,086,377 in Sendai, 53,399 in Shiogama, and 67,566 in Kurihama^1^. The number of older drivers who possessed a car driver’s license in each city was 113,839 in Sendai, 7,574 in Kurihama, and 12,473 in Shiogama^2^. The study protocol was approved by the Ethics Committee of Tohoku University Graduate School of Medicine. This RCT was registered at the University Hospital Medical Information Network (UMIN) Clinical Trial Registry (UMIN000029769).

**Figure 1:**
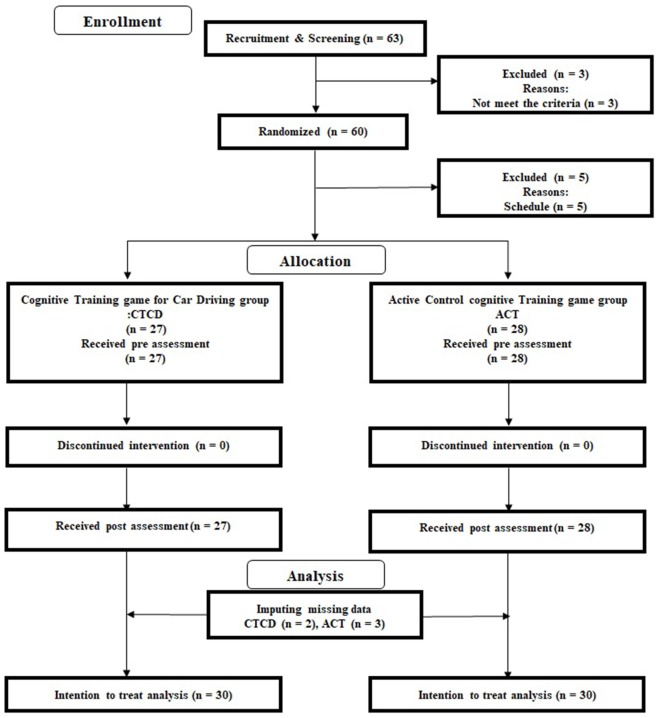
Consolidated Standards of Reporting Trials (CONSORT) diagram.

To assess the benefits of cognitive training on driving performance of healthy older adults, we conducted a double-blinded RCT with an active control group. All participants and testers were blinded to the study hypothesis and the group membership of participants. The primary outcome was driving skills, which was assessed by an on-road evaluation. The secondary outcomes were cognitive functions and emotional states. The Consolidated Standards of Reporting Trials (CONSORT) statement^3^ (see Supplementary Table S1), was used to report the study structure. The RCT design is presented in Figure 1.

### Participants

First, advertisements were used to recruit 60 participants from each of the three cities. We displayed the advertisements at the entrance of the city hall and in small culture schools for 2 weeks. The inclusion and exclusion criteria were printed on the flyers. Sixty-three interested participants contacted the research group by post or by phone (Figure 1) and participated in an orientation meeting. During the meeting, one researcher (RN) explained the study details. We received informed consent from each participant. The researcher then checked whether interested participants were eligible to participate in this study. All participants underwent a cognitive functional screening assessment using the Mini-Mental State Examination (MMSE; Folstein et al., 1975) and Frontal Assessment Battery at bedside (FAB; Dubois et al., 2000). No participant was excluded based on their MMSE and FAB scores. After excluding three participants who did not meet the inclusion criterion of medical history, 60 participants were randomly assigned to the CTCD or ACT group. After the randomization, five participants were excluded because of their schedule. Based on the intention and treatment analysis (ITT), we did not recruit another five participants but imputed the missing data using multiple imputation methods (please see “Analysis” section). Table 1 presents the baseline characteristics of all participants [*n* = 55; 32 men, 23 women; average age = 72.40 years (SD = 3.80)]. There was no significant difference in the baseline data between the two groups (two-sample *t*-test).

**Table 1:** Age, education, driving history, and general cognitive function scores of both groups at baseline.

	CTCD group		ACT group			
	Mean	SD	Mean	SD	Effect size (*d*)	*p*-value
Age (years)	71.67	3.62	73.11	3.90	0.74	0.16
Education (years)	12.96	2.01	12.82	2.04	0.10	0.80
Driving license history (years)	44.54	6.11	45.26	6.26	0.29	0.67
Driving skills (on-road evaluation test score)	113.07	11.75	113.82	9.60	0.23	0.80
MMSE (score)	28.93	1.14	28.96	1.07	0.04	0.90
FAB (score)	15.04	1.45	14.89	1.68	0.12	0.74
JART (score)	113.07	11.75	113.82	9.60	0.23	0.80

*Group comparison (two sample *t*-tests) of pre-training scores revealed no significant difference for any measure (*p* > 0.10). CTCD, Cognitive Training for Car driving Skills group; ACT, Active control group; MMSE, Mini Mental State Examination; FAB, Frontal Assessment Battery at bedside; JART, Japanese Reading Ability Test*.

### Inclusion and Exclusion Criteria

Based on our previous studies (Nouchi et al., 2012a, 2016a,b; Nozawa et al., 2015), we used the following inclusion criteria: (1) right-handed; (2) native Japanese speakers; (3) 65–80 years of age; (4) not concerned about their own memory functions, not using medications known to interfere with cognitive functions (including benzodiazepines, antidepressants, or other central nervous agents); (5) no history of diseases known to affect the central nervous system, including thyroid disease, multiple sclerosis, Parkinson disease, stroke, diabetes, and severe hypertension (systolic blood pressure over 180 mmHg, diastolic blood pressure over 110 mmHg); and (6) daily drivers who have been possessing a valid driver’s license for over 10 years and drive more than thrice a week on average. The exclusion criteria were participants with an MMSE score of less than 26 and FAB score of less than 12. Participants who participated in other cognition-related intervention studies were also excluded.

### Sample Size

We calculated the sample size using a software (Faul et al., 2009). The sample size was based on the change in driving skill score because the primary outcome in this RCT was driving performance. Previous studies applying cognitive training for vehicle operation (Nozawa et al., 2015) described a medium effect size *d* = 0.47 (vs. no intervention group) and *d* = 0.55 (vs. active control group) for driving skill. The pre-post changes in the cognitive training group revealed a large effect size (*d* = 1.13). Thus, we expected an effect size between medium and large (*f* = 0.35). To calculate the sample size, we used an analysis of covariance (ANCOVA) model with pre-intervention driving skill score, sex, and age as covariates, using a one-tailed test, *α* = 0.05, and 0.85 power. The estimated sample size was 60.

### Randomization

To randomly assign the 60 interested participants into the CTCD and ACT groups, we used an online randomization program^4^. We stratified participants based on sex because previous studies reported sex differences in car driving performance as the primary outcome (Ma and Yan, 2014) and cognitive function (McCarrey et al., 2016) and emotional states (Boyle, 1989; Masumoto et al., 2016) as the secondary outcomes. We used blocked randomization (block size: 4) with an allocation ratio of 1:1.

### Overview of the Intervention

Participants were asked to execute the CTCD or ACT protocol at home for 20 min, at least 5 days per week, for a total of 6 weeks (at least 30 training days/sessions and at least a total of 10 h for training). The maximum number of training days was 42 training days/sessions (maximum training hours was 14 h). Previous studies have reported the effects on cognitive performance after self-administered training for 8–10 h on average (Wadley et al., 2006; Ball et al., 2013; Wolinsky et al., 2013).

We used an active control group that had the same training period and a similar training setting. To control for the effects of new experiences such as doing cognitive tasks on a new device, we also developed cognitive training games for an active control group. For the active control group, the difficulty levels of the games did not change during the intervention period. Participants simply completed the cognitive training program at the same level throughout the intervention period. Therefore, cognitive training for the active control group was not intensive adaptive training.

**Figure 2:**
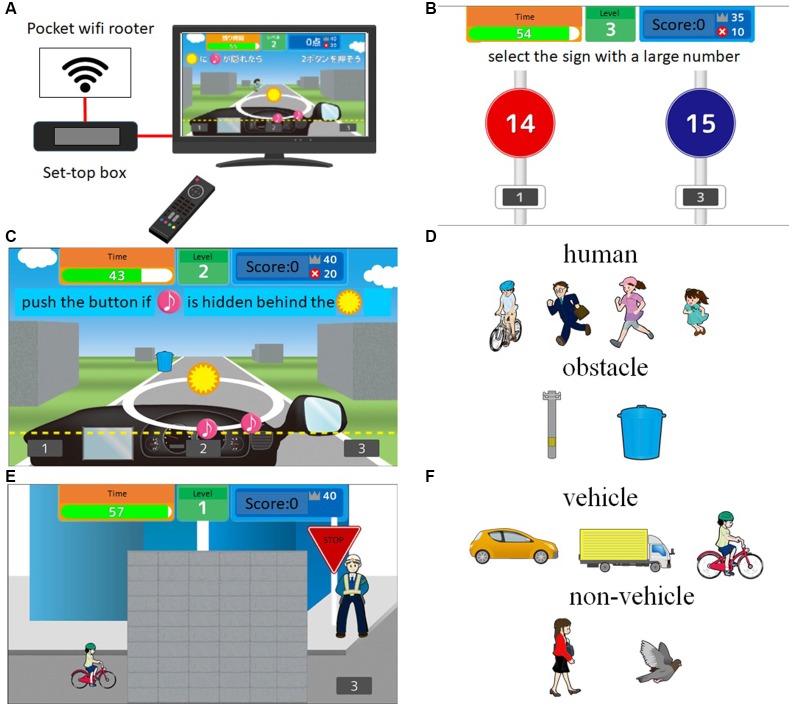
Example of cognitive training games for car driving (CTCD). **(A)** Summary of the intervention setting using the TV and set-top box. (**B**; processing speed training) Two signs with two numbers are presented on the TV screen. Then, participants are asked to select the sign with the large number as quickly as possible **(B)**. In the case shown in **(B)**, the sign on the right is the correct choice. (**C,D**; dual attention training) Participants are asked to perform two tasks simultaneously. For the first task panel **(C)**, the stimulus (pink musical notes) moves along a circle. Then, participants are asked to push the button if the stimulus is hidden behind the mark (orange circle with a yellow star). For the second task, when a target (human or obstacle, see **D**) is approaching, participants are asked to push the button as quickly as possible if the target is human or not push the button if the target is an obstacle. In the case presented in **(C)**, participants do not push the button because the target is an obstacle. (**E**; speed prediction) A target (vehicle or non-vehicle, **F**) is moving through the wall from left to right on TV monitor. Participants are asked to push the button when the target comes out from the wall, but not push the button if the target is a non-vehicle.

The CTCD and ACT training games were played on a TV with a set-top box at home. We provided the set-top box (Hikari BOX, NTT west) and controller, which can be connected to the Internet and enable the user to play a training game (Figure 2A^5^). Before the intervention period, the support staff visited each participant’s home and set up the set-top box, which already had the CTCD or ACT program installed. Participants received instructions on how to use the set-top box and play the training game. All participants played the training game using their own TV. Training game scores and the training duration time were recorded by the set-top box, and the data were automatically transmitted over the internet to our data server using Secure Sockets Layer (SSL)/Transport Layer Security (TLS). Therefore, we were able to verify whether participants completed the training game according to the planned process. Participants were asked to play the training games alone for about 20 min, at least 5 days per week. They were not allowed to lend the set-box to anyone else. We examined the trajectory of the scores in each game to check whether the participant followed the rule. If someone else other than the participant played the game, a gap between the scores in the current play and that in the previous play was observed. We contacted and asked participants to follow the rules if they did not follow rules. At the end of the training period, participants reported their subjective feelings of satisfaction and enjoyment with the training game on a five-point Likert scale: 1, strongly disagree; 2, disagree; 3, neither agree nor disagree; 4, agree; and 5, strongly agree. They also confirmed that they followed the game rules during the intervention period. Then, we confirmed that none of the participants broke the rules. We administered cognitive tests and checked their emotional states 1 day before and after the 6-week intervention period. The set-top box and controller were returned immediately after the completion of the intervention period.

### Cognitive Training Game for Car Driving (CTCD) Group

**Figure 3:**
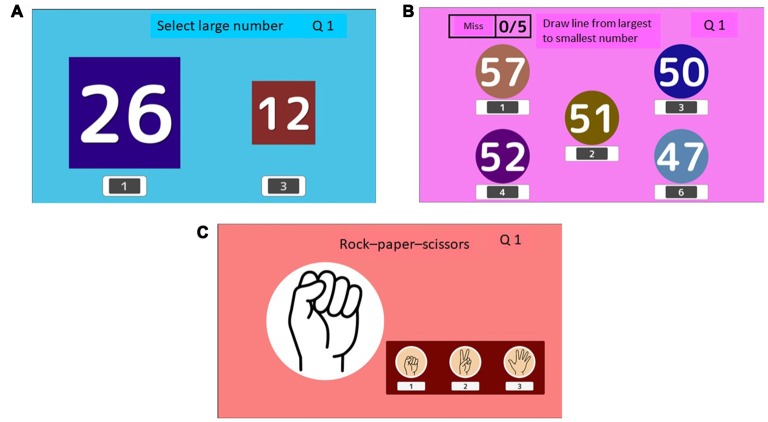
Example of active control cognitive training (ACT). (**A**; select large number) Participants are asked to select the larger number from two numbers presented in different font sizes. In the case shown in **(A)**, the number on the left is the correct answer (26). (**B**; draw a line from the largest to the smallest number) Five numbers are presented. Participants selected numbers in the descending order panel **(B)**. In the case shown in **(B)**, the participant drew a line through numbers 57, 52, 51, 50, and 47. (**C**; rock, paper, scissors) Participants are asked to lose to the hand presented on the computer screen **(C)**. In the case shown in **(C)**, the correct answer is scissors.

We developed three driving-related cognitive training games (processing speed, dual attention, and speed prediction) to be played on the TV and set-top box (Figure 2A). In the processing speed training, two signs with two numbers were presented on the TV screen. Participants were asked to select the sign with the larger number as quickly as possible (Figure 2B). In the case shown in Figure 3A, the sign on the right is the correct choice. In the dual attention training (Figure 2C), participants were asked to perform two tasks simultaneously. For the first task, the stimulus (pink colored musical notes) were moved along a circle. Participants were asked to push the button if the stimulus hid behind the mark (orange circle with yellow star). For the second task, when a target (human or obstacle, see Figure 2D) was approaching, participants were asked to push the button as quickly as possible if the target was human but not push the button if the target was an obstacle. In the speed prediction training (Figure 2E), a target (vehicle or non-vehicle, Figure 2F) moved through the wall from left to right on the TV screen. Participants were asked to push the button when the target came out from the wall but not push the button if the target was a non-vehicle. After finishing each training game, game performance and time were recorded automatically and transmitted to our data server. Therefore, we were able to check whether participants completed the training game based on the planned process. To increase the effects of CTCD, we used an intensive and adaptive training method. The difficulty (level) of each game increased from level 1 to level 20 based on participants’ game performance. To enhance the processing speed elements, we asked the participants to complete all training games as quickly as possible. We developed the cognitive training games to resemble actual driving situations. Participants were asked to play the three training games equally.

### Active Control Cognitive Training Game (ACT) Group (Active Control Group)

We developed three active control cognitive training games (select the larger number; draw a line from the largest to the smallest number; play a game of rock, article, scissors) to be played on the TV and set-top box. In the “select the large number” game (Figure 3A), participants were asked to select the larger number from two numbers displayed in different font sizes. In the case of Figure 3A, the number on the left is the correct answer (26). In the “draw a line from the largest to the smallest number” activity, five numbers were presented. Participants were asked to select the numbers in the descending order (Figure 3B). As shown in Figure 3B, participants drew a line through numbers 57, 52, 51, 50, to 47. In the game of rock, article, scissors, participants were asked to lose to the hand presented on the computer screen (Figure 3C). In the case shown in Figure 3C, the correct answer is scissors. To suppress the elements of processing speed in ACT, we set a fixed time for the answers. The game did not proceed if participants answered quickly. We also required the participants to give the correct answers without rushing. The difficulty level of each game did not increase during the intervention period. Participants simply completed the cognitive training at the same level. Therefore, the ACT was not an intensive adaptive training program. After finishing each game, the game performance and time were recorded automatically and were transmitted to our data server. Participants were asked to play the three training games equally. Consequently, each training game took approximately the same amount of time on each training day.

### Driving Skill

We measured driving skills using an on-road evaluation test (Nozawa et al., 2015), which was conducted at a driving school in Sendai, Japan. All participants used the same car provided by the driving school. Participants drove a car with a driving school instructor for 20 min. Participants completed the driving routes twice. The driving school has a 1.7 km driving course, which includes several curves, signals, hills, and intersections. It was a non-public course. None of the participants were familiar with the routes. To reduce practice and order effects, we prepared four driving routes. The assignment of the driving routes to test orders (first test at pre-intervention, second test at pre-intervention, first test at post-intervention, and second test at post-intervention) was counterbalanced across participants. Participants did not drive the same route. The driving routes had eight driving goals, including a right turn, a left turn, passing a blind intersection (wherein the driver cannot see or had restricted visibility of the traffic coming down the intersecting road), an intersection requiring the driver to stop, change lanes, passing by a stopped car, curve, and other driving decisions and goals. For each driving route, participants drove three laps in the 1.7 km driving course (total 5.1 km). A driving school instructor was seated silently in the passenger seat during the on-roard evaluation test. During the driving task, the instructor checked participants’ driving skill based on the eight driving goals. The on-road evaluation test had 27 checklist items, and the instructor counted behaviors related to the checklist items. The counts on each checklist item were converted into a five-point scale (ranging from 1 to 5). The maximum score was 135, and the minimum score was 27. A higher score represents better driving skills. The driving school instructor had enough experience to evaluate the driving performance of the older adults.

In the pilot study, 20 older adults (mean age = 68.4 years, SD = 2.1) completed the on-road evaluation test twice. The second test was conducted 4 weeks after the first test. The correlation between the two tests was 0.89. The procedure, route, and contents of the car driving evaluation were similar to those of the official car driving test in Japan, indicating that this on-road evaluation test has enough validity.

### Cognitive Functions

To examine the effects of CTCD on cognitive functions, we assessed the scores of processing speed, attention, inhibition, short-term memory, working memory, and episodic memory. Cognitive status was measured using the MMSE and the JART. It took about 1.5 h to complete all the cognitive tests.

To briefly check the cognitive status, we used the MMSE, which measures memory, attention, language, and visuospatial abilities (Folstein et al., 1975). To ascertain participants’ reading ability and IQ, we used the JART (Matsuoka et al., 2006), which is a Japanese version of the National Adult Reading Test (NART). The JART is a reading test consisting of 25 *Kanji* (Chinese characters) compound words. The reading stimuli were randomly printed for reading. Participants were asked to write the pronunciation of each Kanji compound word.

To assess processing speed, we used digit symbol coding (Cd) and symbol search (SS) from the WAIS-III (Wechsler, 1997). The following descriptions of Cd and SS were reproduced from our earlier report (Nouchi et al., 2012b). “For Cd, the participants were shown a series of symbols that were paired with numbers. Using a key within a 120 s time limit, participants drew each symbol under its corresponding number. The primary measure of this test was the number of correct answers. In SS, participants visually scanned two groups of symbols (a target group and a search group) and indicated whether either of the target symbols matched any symbol in the search group. Participants responded to as many items as possible within a 120 s time limit. The primary measure of this test was the number of correct answers.”

To measure attention performance, we conducted the digit cancellation task (D-CAT). The following descriptions of the D-CAT are reproduced from our earlier report (Nouchi et al., 2013). “The test sheet consists of 12 rows of 50 digits. Each row contains five sets of numbers 0–9 arranged in random order. Thus, any one digit would appear five times in each row with randomly determined neighbors. The D-CAT consists of three such sheets. Participants were instructed to search for the target number(s) that had been specified to them and to delete each one with a slash mark as quickly and as accurately as possible until the experimenter sent a stop signal. Three trials were conducted, first with a single target number (6), second with two target numbers (9 and 4), and third with three (8, 3, and 7). Each trial was given 1 min. Consequently, the total time required for D-CAT was 3 min. For the second and third trials, it was emphasized that all instructed target numbers should be canceled without omission. The primary measure of this test was the number of hits (correct answers). We used only the number of hits in the first trial.”

To measure inhibition ability of executive functions, we used a Stroop task (ST) and a reverse Stroop task [rST; Hakoda and Watanabe, 2004; “In the ST, in the leftmost of six columns, a word naming a color was printed in another color (e.g., ‘red’ was printed in blue letters)]; the other five columns contain words naming colors. Participants were required to check the column containing the word naming the color of the word in the leftmost column. In the reverse ST, in the leftmost of six columns, a word naming a color was printed in another color (e.g., ‘red’ was printed in blue letters); the other five columns were filled respectively, with five different colors, from which participants were required to check the column with the color matching the written word in the leftmost column. In each task, participants were instructed to complete as many of these exercises as possible in 1 min. The primary measure for this task was the number of correct items” (Nouchi et al., 2012b).

To measure short-term memory and working memory performance, we used the digit span forward (DS-F) and the digit span backward (DS-B) tasks, which are the subtests of the WAIS-III (Wechsler, 1997). The following descriptions of the DS-F and the DS-B are reproduced from our earlier report (Nouchi et al., 2012b). “For the DS-F, participants repeated numbers in the same order as they were read aloud by the examiner. For the DS-B, participants repeated numbers in the reverse order of that presented aloud by the examiner. In both tasks, the examiner read a series of number sequences which the participant was required to repeat in either forward or reverse order.” The primary measures of this test were the digit number length. The maximum digit number length in the DS-F was 8, and that of the DS-B was 7.

To measure episodic memory, we used the logical memory (LM) subtest of the WMS-R (Wechsler, 1987): “LM consists of two short-paragraph-length stories (Story A and Story B). For the LM activity, participants were required memorize one of the two stories. The stories were scored in terms of the number of story units recalled, as specified in the WMS-R scoring protocol. We used either Story A or Story B. The primary measure for this task was the number of correct story units recalled” (Nouchi et al., 2012b). We checked the performances of immediate recall and delayed recall memory.

### Emotional State Measure

To assess the change in emotional state, we used a short version of the Profile of Mood State Second Edition (POMS2; Heuchert and McNair, 2012; Yokoyama and Watanabe, 2015). The POMS2 has 35 items rated on a 5-point scale. These items are divided into POMS, which has seven subscales with 5-point scales (total 35 items). The POMS2 can measure the following emotional states in the prior week: Tension–Anxiety (T–A), Depression–Dejection (d), Anger–Hostility (A–H), Vigor–Activity (V), Fatigue–Inertia (F–I), Confusion–Bewilderment (c), and Friendliness (F).

### Analysis

All participants were included based on the intention to treat (ITT) principle. First, we calculated the change scores in driving skills, cognitive functions, and emotional states (post-intervention score minus pre-intervention score). Second, we imputed missing data using the multiple imputation method (predictive mean matching, *m* = 20). All variables of the pre-, post-, and change scores and participants’ physical information (age, sex) were included in the data imputation process. We performed multiple imputations using the function of “mice” in the mice package (van Buuren and Groothuis-Oudshoorn, 2011). Third, using all 20 imputed datasets, we performed an ANCOVA with permutation tests for all change scores. We performed permutation tests because they are suitable for small sample analysis and are distributed freely. We used a one-tailed test because we had a strong hypothesis that the CTCD program would have more positive effects than would the ACT program. In the ANCOVA, the change score was considered as the dependent variable. The group was considered as the independent variable. The pre-scores in the dependent variable, MMSE, age, and sex were used as covariates. All ANCOVAs with the permutation test were performed using the “aovp” function in the lmPerm package^6^. Fourth, we combined/pooled the *F* values from all results of the 20 imputed datasets using the “micombine.F” function in the “miceadds” package^7^. The method of pooling the *F* Value was based on previous studies (van Buuren and Groothuis-Oudshoorn, 2011; Grund et al., 2016). Finally, we used false discovery rate (FDR) correction methods to adjust all pooled *p* values (Benjamini and Hochberg, 2000). All analyses were performed using software (R ver. 3.50).

For the additional analysis, if we detected improvement in driving skill, we performed a permutation multiple regression analysis of improvements in driving skills, cognitive functions, and mood. The additional analysis was intended to elucidate the relationship between changes in driving skill and changes in cognitive functions, and changes in moods. The pre-score in the dependent variable, MMSE score at baseline, age, and sex were used as covariates. After scaling all imputed datasets using the “mids2datlist” and “scale_datlist” functions in the “miceadds” package, we performed permutation multiple regression analyses using the “lmp” function in lmperm package. Finally, we combined/pooled all results from the 20 imputed datasets using Rubin’s rule (Barnard and Rubin, 1999; Rubin, 2004). Significance was inferred for *p* < 0.05 for multiple comparison methods.

## Results

There was no significant difference in the average number of training days between CTCD (*M* = 32.04 days, SD = 2.25) and ACT (*M* = 31.57 days, SD = 1.93) groups and in the total training hours between CTCD (*M* = 10.61 h, SD = 1.19) and ACT (*M* = 10.17 h, SD = 1.03) groups. The maximum game levels for the CTCD group were Game 1 (*M* = 18.46, SD = 3.50), Game 2 (*M* = 13.96, SD = 6.42), and Game 3 (*M* = 11.38, SD = 3.63). We evaluated participants’ satisfaction and enjoyment after the intervention using a five-point scale. There was no significant difference in the average satisfaction scores between CTCD (*M* = 3.83, SD = 0.54) and ACT (*M* = 3.78, SD = 0.43) groups or and in enjoyment scores between CTCD (*M* = 3.92, SD = 0.71) and ACT (*M* = 3.88, SD = 0.83) groups. There was no significant difference between the two groups with respect to the measures at baseline (Table 1). The cognitive functions and emotional states after the intervention period was shown in Supplementary Table S2.

Three participants in the CTCD group and two in the ACT group dropped out during the intervention period because of their respective schedules. By the intention-to-treat rule, we imputed missing values of the five participants (please see “Analysis” section). To check the benefits of CTCD on driving skills, cognitive functions, and emotional state, we performed a permutation test with ANCOVAs for the change scores (Tables 2, 3). In terms of driving skill measures, the CTCD group showed a significant improvement (*F*_(1,469.88)_ = 7.987, η^2^ = 0.08, *adjusted p* = 0.034). Regarding cognitive abilities, the CTCD group showed a significant improvement in processing speed performance with respect to the Cd score (*F*_(1,567.29)_ = 5.161, η^2^ = 0.10, *adjusted p* = 0.047) and the SS score (*F*_(1,678.65)_ = 6.63, η^2^ = 0.09, *adjusted p* = 0.049). Furthermore, the CTCD group showed a significant improvement in inhibition performance with respect to the rST score (*F*_(1,8006.18)_ = 5.061, η^2^ = 0.06, *adjusted p* = 0.043) and the ST score (*F*_(1,2360.02)_ = 3.945, η^2^ = 0.06, *adjusted p* = 0.042). Regarding the emotional state, the CTCD group showed improvement in the V–A score in POMS (*F*_(1,655.54)_ = 4.46, η^2^ = 0.06, *adjusted p* = 0.041). There was no significant difference in other measures. In summary, the CTCD group demonstrated improvements in driving skills, two cognitive domains (processing speed and inhibition), and emotional state (vigor–activity).

**Table 2:** Cognitive function and emotional states scores of both groups at baseline.

	CTCD group		ACT group			
	Mean	SD	Mean	SD	Effect size (*d*)	*p*-value
Processing speed
Cd (number)	61.48	10.36	58.07	10.70	1.05	0.24
SS (number)	30.81	2.94	29.18	5.13	0.81	0.15
Executive functions (inhibition)						
rST (number)	38.85	5.43	36.14	5.75	1.15	0.08
ST (number)	27.41	7.63	23.93	8.02	1.24	0.11
Short-term memory						
DS-F (digit number)	5.30	1.03	5.18	1.12	0.11	0.69
Working memory						
DS-B (digit number)	4.04	1.13	3.89	0.83	0.15	0.59
Attention						
D-CAT (number)	4.04	1.13	3.89	0.83	0.15	0.59
Episodic memory						
LM immediate (score)	8.74	4.13	9.07	3.53	0.17	0.75
LM delay (score)	8.26	3.90	8.54	3.71	0.14	0.79
Emotional states						
T–A in POMS (score)	4.22	2.36	4.00	3.15	0.13	0.77
D in POMS (score)	1.67	1.98	1.93	2.16	0.18	0.64
A–H in POMS (score)	2.30	2.25	1.68	1.93	0.43	0.28
V in POMS (score)	8.59	4.00	7.64	3.49	0.49	0.35
F_I in POMS (score)	8.59	4.00	7.64	3.49	0.49	0.35
C in POMS (score)	2.41	1.97	2.50	2.40	0.06	0.88
F in POMS (score)	12.00	4.45	11.04	3.83	0.47	0.39

**Table 3:** Change scores of cognitive function and emotional states of both groups.

	CTCD group		ACT group				
	Mean	SD	Mean	SD	Effect size (η^2^)	adjusted *p*-value	non-adjusted *p-*value
Car driving skill							
On-road evaluation	7.07	11.21	0.10	9.89	0.08	0.034	0.002
Processing speed							
Cd (number)	4.48	4.64	0.68	5.56	0.10	0.049	0.010
SS (number)	3.15	3.16	0.79	3.67	0.09	0.047	0.007
Executive functions (inhibition)							
rST (number)	4.00	4.88	0.89	5.01	0.06	0.043	0.015
ST (number)	2.70	3.23	0.29	4.48	0.06	0.042	0.012
Short-term memory							
DS-F (digit number)	0.00	1.39	0.29	1.01	0.02	0.273	0.156
Working memory							
DS-B (digit number)	0.33	0.96	0.21	0.99	0.00	0.323	0.369
Attention							
D-CAT (number)	12.37	25.13	11.14	17.79	0.00	0.314	0.381
Episodic memory							
LM immediate (score)	0.48	3.32	−0.18	2.84	0.01	0.293	0.209
LM delay (score)	0.33	3.06	−0.32	3.32	0.01	0.277	0.218
Emotional states							
T–A in POMS (score)	0.37	2.24	−0.04	2.20	0.01	0.290	0.269
D in POMS (score)	0.15	2.21	0.07	2.39	0.00	0.327	0.420
A–H in POMS (score)	0.30	1.49	−0.07	2.00	0.01	0.286	0.286
V in POMS (score)	2.59	3.20	0.71	3.22	0.06	0.041	0.018
F_I in POMS (score)	−0.19	2.62	−0.86	1.96	0.02	0.316	0.143
C in POMS (score)	0.44	1.91	−0.07	2.34	0.01	0.305	0.262
F in POMS (score)	−0.59	2.45	0.07	2.79	0.01	0.316	0.203

Additionally, to investigate the relationships among these improved skills, we separately performed multiple regression analyses for the CTCD and ACT groups. The covariates were age, sex, MMSE at baseline, and the pre-score on the dependent variable. The results of the CTCD group showed a significant positive correlation between improved driving skill and improved Cd score (*standardized* *β* = 0.55, *t* = 2.76, *p* = 0.01) and between improvements in driving skills and SS score (*standardized* *β* = 0.63, *t* = 3.65, *p* = 0.00). There was no significant correlation between the improved driving skills or other improvements. Moreover, no significant result was found in the multiple regression analyses of the ACT group.

## Discussion

We developed new cognitive training games to improve the driving skills in older adults. The cognitive training games can be played on a TV using a set-top box. We investigated the benefits of CTCD on driving skills, cognitive functions, and emotional states of healthy older people. Our study revealed three main findings. First, the results of the on-road evaluation tests showed that the 6-week CTCD intervention improved participants’ driving skills. Additionally, we found a significant positive correlation between the change scores of driving skills and the change scores of processing speed performance. Second, the CTCD group demonstrated improved cognitive performances in terms of processing speed measured by Cd and SS and inhibition performance measured by ST and rST compared to ACT. Third, CTCD showed improvement in vigor–activity mood, measured using the POMS. Taken together, the results of this study extend those of previous studies by demonstrating improvements in driving skills, cognitive functions, and emotional states. These findings are discussed below.

The first main finding is that the CTCD program improved the driving skills of healthy older adults. Our result is consistent with those of previous studies using cognitive training for driving skill (Edwards et al., 2009b; Haeger et al., 2018; Ross et al., 2018). For example, an 8-week cognitive training in a laboratory showed improvement in driving skills of the older adults (Nozawa et al., 2015). A unique aspect of the present study is that our cognitive training programs were conducted with a TV at home. Almost all cognitive training for driving skills was conducted at the laboratory with trainers or using specific devices (Casutt et al., 2014a, 2016; Nozawa et al., 2015; Haeger et al., 2018). This suggests that driving skills in older adults can be improved through cognitive training both at home and at the laboratory. It is important to note the significance of the change in scores of the car driving performance. Car driving skills were measured by an on-road evaluation test. A higher score indicates higher car driving skills. The average score was 113 (84%) out of 135, indicating that our participants had good car driving skills. The effect size (η^2^) was 0.08, indicating that our intervention had a medium effect for improvements of car driving skills in healthy older adults. However, it does not mean that individuals who scored high in this car driving test are safe drivers. From the current result, it is difficult to conclude that the cognitive training can improve car driving safety. Therefore, in the future, it is important to examine whether cognitive training reduces car accidents. This study did not verify the long-term benefits after cognitive training. We measured driving skills immediately after the training period. Therefore, we demonstrated immediate training effects of cognitive training on driving skills. Our short-term cognitive training might not have long-term benefits for driving skills. To clarify the current results, future studies should assess long-term benefits using follow up assessments for several years. It is particularly interesting that we found a positive correlation between improvements of driving skills and improvements of cognitive functions, which suggest that changes in driving skills and cognitive functions share the same mechanism of performance improvement, which is discussed later.

The second main result of the present study is that the CTCD program improved the processing speed and inhibition of executive functions. For processing speed, previous studies of cognitive training for driving also showed significant improvement in processing speed, measured using Cd (Hay et al., 2016), simple reaction time (Casutt et al., 2014b), and the composite processing speed score, which combines the TMT-A and Cd scores (Nozawa et al., 2015). Supporting the existing evidence, we found improvement in processing speed as measured by Cd. Additionally, we expanded the existing evidence for improvement in processing speed, measured by SS. Nevertheless, it remains unclear whether cognitive training can improve other processing speed measures, such as UFOV. Previous studies have demonstrated that cognitive training for driving skills improves UFOV (Roenker et al., 2003). To generalize our findings, it is important to assess cognitive training improvements in UFOV, if any.

Regarding executive functions, previous studies using cognitive training for driving skills also showed improvements in shifting performance of executive functions, measured using Trail Making Test B (Hay et al., 2016). Our results are the first to demonstrate an improvement in the inhibition of executive functions, measured using ST and rST, after a 6-week cognitive training. It is noteworthy that a previous study using cognitive training for driving skills (Casutt et al., 2016) failed to demonstrate an improved inhibition performance, measured by ST. Several methodological differences exist between the current study and the previous study (Casutt et al., 2016). For example, although the previous study’s cognitive training program focused on only a single cognitive domain such as attention, our cognitive training focused on multiple cognitive domains, such as processing speed, inhibition, and attention. previous studies have reported that multiple domain training has more benefits than does single-domain training (Cheng et al., 2012). The evaluation of the performance on the ST differed between the current study (the number of correct responses) and the previous study (reaction time). Additionally, we used the article version of the ST, whereas the previous study used the PC version of ST along with EEG recordings. These methodological differences might affect the inconsistent results. Future studies should use similar administration and scoring methods for the ST.

The CTCD group in the present study showed no significant improvement in attention or memory performance. These results are consistent with those of previous studies using short-term cognitive training for driving skills. For attention performance, previous studies reported no significant improvement in sustained, selective, or divided attention performance (Casutt et al., 2014b). However, a long-term intervention study using cognitive training for driving skills found improvement in the performance of choice reaction time (Roenker et al., 2003). One possibility is that the 6-week intervention period is insufficient to improve the attention performance. For memory performance, a previous study measured short-term memory using the Benton Visual Retention test, working memory using the spatial span test, and episodic memory using the Rey auditory–verbal learning test (Nozawa et al., 2015). The study found no significant improvement in the composite memory score included all memory performance (Nozawa et al., 2015). In keeping with the findings of previous studies, we found no significant improvements in other memory measures, such as the digit span for short-term and working memory performance and LM for episodic memory. An explanation for this finding is that memory was not targeted in our cognitive training. In addition, previous studies of cognitive training for driving skills did not specifically use memory measures. A few studies have measured memory performance. Therefore, we cannot infer that cognitive training for driving skills has no beneficial effects on memory performance. Future studies should be conducted to measure multiple memory performance better. It will be possible to verify the benefits of cognitive training on driving skills in terms of memory performance.

Improvements in driving skills, processing speed, and inhibition are explained by the overlapping hypothesis (Nouchi and Kawashima, 2014; Nouchi et al., 2016a). The overlapping hypothesis assumes that driving skills and cognitive functions will improve with cognitive training when training tasks and untrained measures share common mental processes (Nouchi et al., 2016a). Based on the Cattel–Horn–Carroll (CHC) model (Schneider and McGrew, 2012), behavioral and mental processes can be divided into three levels, narrow, broad, and general abilities: “the broad ability of processing speed consists of several narrow abilities such as perceptual speed, rate of test-taking, number facility, reading speed, and writing speed” (Nouchi et al., 2016a). In fact, driving skill is a general ability that includes some narrow and broad abilities. Previous studies have reported that driving skills are correlated with several cognitive functions such as processing speed (Anstey et al., 2005; Horikawa et al., 2009; Adrian et al., 2011). Therefore, we infer that driving skills consist of several broad abilities, such as cognitive processing and executive functions. In this study, participants were asked to complete three training tasks (processing speed, dual attention, and speed prediction) during the 6-week intervention period. All training tasks involved processing speed and inhibition components. For example, we asked participants to complete the training games as quickly as possible. Therefore, processing speed is a common factor in the completion of all training tasks. Inhibition processes also play an important role in selecting appropriate targets of the processing speed game, by ignoring distractors in the dual attention game, pushing the button at an appropriate timing in the speed prediction game, and following the appropriate rules in each training game. Based on the overlapping hypothesis, improvements in driving skill, processing speed, and inhibition performance are sufficient to explain the following notion. Training games require mental processes including processing speed and inhibition components. The cognitive training game, the measurement of driving skills, and the measurements of cognitive functions shared similar mental process. Mental processes related to processing speed and inhibition were used in the training games. Although playing cognitive training games, these mental processes are expected to be facilitated and enhanced. Therefore, processing speed and inhibition were improved directly because all gaming tasks required processing speed and inhibition processes. Furthermore, driving skills improved because processing speed and inhibition processes were involved in the driving skills.

The third main finding of the present study is the improvement in vigor mood in POMS. A previous study demonstrated that long-term processing speed training for healthy older adults leads to reduction of depressive symptoms, measured by the Center for Epidemiologic Studies Depression Scale (CES-D; Wolinsky et al., 2009). A short-term cognitive training study of healthy older adults showed a change in depressive mood, measured by the POMS (Nouchi et al., 2016a). However, our study showed no depressive mood reduction. One explanation for this might be that our participants reported lower depressive scores at baseline (the average depression score was less than 2), indicating that our participants did not experience depressive symptoms. Therefore, we found no reduction in the depressive mood in this study. However, this study is the first to demonstrate an improvement in vigor mood in healthy older adults after a 6-week cognitive training for driving skills.

A potential mechanism underlying improved vigor mood might be that the cognitive training functioned as emotional regulation. A previous study demonstrated that cognitive tasks reduce negative emotions (Iida et al., 2011, 2012). A neuroimaging study using cognitive training also found that brain activity in the insula for emotional stimuli was reduced during the 4-week cognitive training (Takeuchi et al., 2014). In addition, several previous studies reported that playing video games can induce a positive mood (Russoniello et al., 2009; Granic et al., 2014; Pallavicini et al., 2018). Based on these findings, we infer that cognitive training might induce the person to ignore negative experiences and experience a positive mood in the cognitive training. Participants in this study experienced positive emotions during the training because the difficulties in the cognitive training games changed their game performance. Therefore, vigor emotion increased following the cognitive training intervention. To generalize these findings, we need to apply the same intervention to other populations (young adults or a clinical population). Moreover, if we could use a neuroimaging technique, such as functional magnetic resonance imaging (fMRI), we can provide new insights into the neural basis of emotional states after cognitive training.

Our study has some advantages over previous studies. First, to familiarize the general public with cognitive training, developing user-friendly cognitive training tools is a key step. In this study, we developed the cognitive training games to be played on a TV and then presented scientific evidence. Cognitive training using a TV is an effective and easy approach to conduct cognitive training for older people at any time because they usually own a TV at home and know how to operate it. Therefore, we believe that our study provides a new, useful, and effective tool for cognitive training. Second, we controlled for the effect of performing a regular cognitive task using active control games. In these active control games, participants were asked to do three cognitive training games that included some cognitive functions. However, the level of difficulty of the game did not change, suggesting that doing multiple cognitive training is not important to improve cognitive performance. An intensive and adaptive approach, whereby the task difficulty changes based on participants’ performance, provides more key elements to improve the performance. To elucidate the key elements of the CTCD, future studies should be conducted with RCT using non-adaptive CTCD as an active control group.

This study has some limitations. First, this study investigated whether a 6-week short-term training can improve driving skills, cognitive functions, and emotional states of healthy older people. A cognitive training study of a large sample demonstrated that the benefits of 1-year processing speed training program were observed for 5 years (Willis et al., 2006). It is, therefore, important to investigate whether the benefits of this intervention program are lasting. Second, we measured driving skills based on driving performance at a driving school. This method has several salient benefits. For example, it can readily control traffic conditions and measure basic driving skills. Therefore, we can measure driving skills in the same situation for all participants. However, it is also important to check driving behaviors in everyday situations. Future studies should measure driving skills during several situations, such as a traffic jam or a bad weather condition or during night-time driving. Generalizing the effects of a short-term cognitive training program on driving skills requires further verification of the benefits of the short-term processing speed training on long-lasting effects and everyday driving behaviors. Finally, we only evaluated the benefits of cognitive training on car driving skills, cognitive function, and emotional state. However, it remains unclear whether our cognitive training program can improve car driving safety in older adults, which can reduce car accidents among older adults. Future studies should investigate these important issues.

## Conclusion

In conclusion, we developed a TV-based CTCD program and conducted an RCT to assess the benefits of a 6-week CTCD on driving skills, cognitive functions, and mood states in older car drivers. Our study provides scientific evidence that the CTCD program improves driving skills, processing speed, inhibition performance, and vigor–activity mood. Our results extend previous findings which demonstrated the benefits of cognitive training on driving skills, processing speed, inhibition, and vigor–activity mood in older people.

## Ethics Statement

Ethical approval was provided by the Institutional Review Board of the Tohoku University Graduate School of Medicine (2017-2-209-1). This study was conducted according to the principles outlined in the Declaration of Helsinki. Written informed consent was received from each participant.

## Author Contributions

RN designed and developed the study protocol and analyzed all data. RN, AK, and HN conducted the study. RN wrote the manuscript with AK, HN, and RK. RK provided advice related to the study protocol. All authors have read and approved the final manuscript.

## Conflict of Interest Statement

RK developed the cognitive training games with Sendai Television Inc., which also provided a set-top box with training games and a portable Wi-Fi to participants. After this study, all devices were returned to Sendai Television Inc. This study was thereby supported to that extent by Sendai Television Inc. Nevertheless, sources of funding for this study had no involvement in the study design, collection, analysis, interpretation of data, or writing of the manuscript. The remaining authors declare that the research was conducted in the absence of any commercial or financial relationships that could be construed as a potential conflict of interest.

## Footnotes

^1^https://www.pref.miyagi.jp/uploaded/attachment/653161.pdf

^2^http://www.police.pref.miyagi.jp/hp/menkyo/mennkyo_%20toukei.pdf

^3^http://www.consort-statement.org/home/

^4^http://www.graphpad.com/quickcalcs/index.cfm

^5^https://www.ntt-west.co.jp/kiki/hikaribox/hb-2000/spec/

^6^http://cran.r-project.org/web/packages/lmPerm/index.html

^7^https://github.com/alexanderrobitzsch/miceadds
