# Supplementary material for: Newly Developed TV-Based Cognitive Training Games Improve Car Driving Skills, Cognitive Functions, and Mood in Healthy Older Adults: Evidence From a Randomized Controlled Trial
Source: Front Aging Neurosci. 2019 May 7;11:99. doi: 10.3389/fnagi.2019.00099 (PMC6513888; doi:10.3389/fnagi.2019.00099)
Supplement: Supplementary file 2 [file Table_2.DOCX]

**Supplementary Table 1. Cognitive function and emotional states scores after intervention in both groups**

|  | CTCD group | | ACT group | |  |  |
| --- | --- | --- | --- | --- | --- | --- |
|  | Mean | SD | Mean | SD | Effect  size (*d*) | *p*-value |
| Car driving skill  On-road evaluation test  Processing speed | 120.15 | 3.92 | 113.93 | 10.46 | 2.31 | 0,01 |
| Cd (number) | 65.96 | 12.39 | 58.75 | 11.76 | 2.08 | 0.03 |
| SS (number) | 33.96 | 4.87 | 29.96 | 5.61 | 1.75 | 0.01 |
| Executive functions (inhibition) |  |  |  |  |  |  |
| rST (number) | 42.85 | 6.99 | 37.04 | 7.94 | 2.13 | 0.01 |
| ST (number) | 30.11 | 7.24 | 24.21 | 8.41 | 2.11 | 0.01 |
| Short-term memory |  |  |  |  |  |  |
| DS-F (digit number) | 5.30 | 1.35 | 5.46 | 1.14 | 0.15 | 0.62 |
| Working memory |  |  |  |  |  |  |
| DS-B (digit number) | 4.37 | 1.11 | 4.11 | 0.99 | 0.26 | 0.36 |
| Attention |  |  |  |  |  |  |
| D-CAT (number) | 152.59 | 28.06 | 137.54 | 30.92 | 2.77 | 0.06 |
| Episodic memory |  |  |  |  |  |  |
| LM immediate (score) | 9.22 | 3.52 | 8.89 | 2.95 | 0.18 | 0.71 |
| LM delay (score) | 8.59 | 3.47 | 8.21 | 2.75 | 0.21 | 0.66 |
| Emotional states |  |  |  |  |  |  |
| T-A in POMS (score) | 4.59 | 2.96 | 3.96 | 2.95 | 0.37 | 0.43 |
| D in POMS (score) | 1.81 | 2.47 | 2.00 | 1.94 | 0.12 | 0.76 |
| A-H in POMS (score) | 2.59 | 2.19 | 1.61 | 2.15 | 0.67 | 0.10 |
| V in POMS (score) | 11.19 | 2.47 | 8.36 | 4.14 | 1.55 | 0.00 |
| F_I in POMS (score) | 2.30 | 3.11 | 2.00 | 2.13 | 0.18 | 0.68 |
| C in POMS (score) | 2.85 | 2.78 | 2.43 | 2.52 | 0.26 | 0.56 |
| F in POMS (score) | 11.41 | 4.05 | 11.11 | 4.05 | 0.15 | 0.78 |
